# Barriers and facilitators to participation in exercise prehabilitation before cancer surgery for older adults with frailty: a qualitative study

**DOI:** 10.1186/s12877-023-03990-3

**Published:** 2023-06-06

**Authors:** Keely Barnes, Emily Hladkowicz, Kristin Dorrance, Gregory L. Bryson, Alan J. Forster, Sylvain Gagné, Allen Huang, Manoj M. Lalu, Luke T. Lavallée, Chelsey Saunders, Hussein Moloo, Julie Nantel, Barbara Power, Celena Scheede-Bergdahl, Monica Taljaard, Carl van Walraven, Colin J. L. McCartney, Daniel I. McIsaac

**Affiliations:** 1 Clinical Epidemiology Program, Ottawa Hospital Research Institute, Ottawa, Canada; 2 Department of Anesthesiology and Pain Medicine, University of Ottawa and The Ottawa Hospital, The Ottawa Hospital Civic Campus, Room B311, 1053 Carling Ave, Ottawa, ON K1Y 4E9 Canada; 3 Division of General Internal Medicine, University of Ottawa and The Ottawa Hospital, Ottawa, Canada; 4 Division of Geriatric Medicine, University of Ottawa and The Ottawa Hospital, Ottawa, Canada; 5 Division of Urology, University of Ottawa and The Ottawa Hospital, Ottawa, Canada; 6 Division of General Surgery, University of Ottawa and The Ottawa Hospital, Ottawa, Canada; 7 School of Human Kinetics, Faculty of Health Sciences, University of Ottawa, Ottawa, Canada; 8 Department of Kinesiology and Physical Education, McGill University, Montreal, Canada; 9 School of Epidemiology & Public Health, University of Ottawa, Ottawa, Canada; 10 Department of Medicine, University of Ottawa and The Ottawa Hospital, Ottawa, Canada

**Keywords:** Prehabilitation, Frailty, Qualitative methods

## Abstract

**Background:**

Older adults with frailty are at an increased risk of adverse outcomes after surgery. Exercise before surgery (exercise prehabilitation) may reduce adverse events and improve recovery after surgery. However, adherence with exercise therapy is often low, especially in older populations. The purpose of this study was to qualitatively assess the barriers and facilitators to participating in exercise prehabilitation from the perspective of older people with frailty participating in the intervention arm of a randomized trial.

**Methods:**

This was a research ethics approved, nested descriptive qualitative study within a randomized controlled trial of home-based exercise prehabilitation vs. standard care with older patients (≥ 60 years) having elective cancer surgery, and who were living with frailty (Clinical Frailty Scale ≥ 4). The intervention was a home-based prehabilitation program for at least 3 weeks before surgery that involved aerobic activity, strength and stretching, and nutritional advice. After completing the prehabilitation program, participants were asked to partake in a semi-structured interview informed by the Theoretical Domains Framework (TDF). Qualitative analysis was guided by the TDF.

**Results:**

Fifteen qualitative interviews were completed. Facilitators included: 1) the program being manageable and suitable to older adults with frailty, 2) adequate resources to support engagement, 3) support from others, 4) a sense of control, intrinsic value, noticing progress and improving health outcomes and 5) the program was enjoyable and facilitated by previous experience. Barriers included: 1) pre-existing conditions, fatigue and baseline fitness, 2) weather, and 3) guilt and frustration when unable to exercise. A need for individualization and variety was offered as a suggestion by participants and was therefore described as both a barrier and facilitator.

**Conclusions:**

Home-based exercise prehabilitation is feasible and acceptable to older people with frailty preparing for cancer surgery. Participants identified that a home-based program was manageable, easy to follow with helpful resources, included valuable support from the research team, and they reported self-perceived health benefits and a sense of control over their health. Future studies and implementation should consider increased personalization based on health and fitness, psychosocial support and modifications to aerobic exercises in response to adverse weather conditions.

**Supplementary Information:**

The online version contains supplementary material available at 10.1186/s12877-023-03990-3.

## Introduction

Older adults (i.e., ≥ 65 years) are a rapidly growing demographic and represent the majority of people who have major surgery [1, 2]. Four out of ten older adults who have surgery also live with frailty [3], which is defined as a state related to loss of reserve across multiple domains leading to vulnerability to adverse health outcomes [4, 5]. Approximately one in five older people with frailty experience a new patient-reported disability after surgery [3]. Frailty is further associated with a twofold or greater increase in the risk of postoperative morbidity, mortality and institutional discharge [6–10]. As the population ages, the number of older people with frailty having surgery, and experiencing adverse events is expected to grow [10].

Exercise prehabilitation (i.e., exercise therapy performed before surgery) has been identified as a promising intervention to address the vulnerability to physical and physiological stressors (disability, falls, delirium, serious illness, hospitalization) that individuals with frailty encounter [5, 11–13]. In fact, evidence suggests that prehabilitation may be most effective in older people with frailty or frailty characteristics [12, 14]. Frailty characteristics can include feelings of fatigue or low energy, loss of strength, or physical limitations/comorbidities that impact daily activities [5]. However, average adherence with exercise therapy is 70% [15], and is even lower among older populations with frailty (~ 60%) [16–18]. Prehabilitation trials in older people with frailty suggest high levels of efficacy in adherent participants, but have failed to show impact on outcomes across all participants [17, 18]. Without strong adherence, it is unlikely that future prehabilitation interventions will prove effective in improving outcomes. While there is variability in the location of prehabilitation interventions (home, outpatient clinics, in-hospital, combined) [15], older adults value home-based care [19] and there is evidence to suggest increased adherence to home-based prehabilitation [20]. Currently, there is limited understanding of what drives adherence to prehabilitation. Few patient-reported data are available to provide insights into participation in prehabilitation from the perspectives of older adults [21].

Theory-based behaviour change frameworks can be used to design interventions to improve adherence to target health behaviours, such as prehabilitation. A leading behavior change framework is the Theoretical Domains Framework (TDF) [22], which can be used to identify barriers and facilitators to patient’s health behaviors. Therefore, to inform future design and implementation of exercise prehabilitation programs in older people with frailty, we conducted TDF-informed interviews with older adults with frailty participating in a home-based prehabilitation program before elective cancer surgery. We aimed to identify the barriers and facilitators of participating in exercise prehabilitation from the perspective of older adults with frailty who had recently completed their prescribed prehabilitation program.

## Materials & methods

### Study design and setting

This was a nested descriptive qualitative study [23, 24] within a single center, parallel-arm, randomized controlled trial of home-based exercise prehabilitation (the PREHAB Study) [18, 25]. Results of the randomized trial are reported elsewhere, but in brief there was no strong evidence for intervention efficacy in improving postoperative functional recovery across all participants (6 min walk test distance + 14 m, 95%CI -26 to 55), but a large, positive effect among adherent participants (+ 76 m, 95%CI 30 to 122). The PREHAB Study and nested descriptive qualitative study were conducted at The Ottawa Hospital (TOH), a 900-bed tertiary care academic health sciences center serving a catchment area of 1.2 million people as the regional cancer referral center for the Eastern portion of the Canadian province of Ontario. On average, intraabdominal, intrathoracic and pelvic cancer patients are seen 4 weeks prior to surgery (as this is a provincial benchmark for cancer care). Ethics approval was granted by The Ottawa Health Sciences Network – Research Ethics Board (OHSN-REB Protocol #20160091). The ‘Consolidated criteria for reporting qualitative research (COREQ): a 32-item checklist for interviews and focus groups’ [26] can be found in Additional File 1.

### Theoretical frameworks

This qualitative research study was situated with the Social Constructivist [27] paradigm where it is believed that there are multiple truths and realities which are created and developed through social interactions. Within this framework, it is important to acknowledge the backgrounds and contexts of the researchers [27].

The TDF was used to guide the interviews, while recognizing that there is not one objective truth. The TDF is a behaviour change framework that comprises 14 theoretical domains derived from 128 constructs from a synthesis of 33 different theories of health, behavioural and social psychology that explain changes in health-related behaviour [22, 28]. The framework was developed by health psychology theorists, health services researchers and health psychologists. While the TDF was originally designed for studying health professional behavior change, it has been applied to several patient behavior change scenarios, including physical activity [29, 30].

### Reflexivity of the research team

The research team included the Principal Investigator, a male Anesthesiologist and health services researcher with expertise in perioperative medicine and frailty (DIM), a female PhD candidate in Aging & Health with a Masters degree in Counselling Psychology and experience with prehabilitation for older adults (EH), and two female clinical research assistants, one with a Masters in Human Kinetics and experience with the TDF (KD) and one with a Masters in Exercise Psychology and experience with qualitative interviews (CS).

### Participants and recruitment

Patients were eligible to participate in the main PREHAB Study if they were ≥ 60 years of age, lived with frailty based on the Clinical Frailty Scale (CFS, a 9-point global frailty scale based on clinical evaluation and judgement of an individual’s mobility, energy, physical activity and function [4].; score of ≥ 4/9), were able to communicate in French or English, and were scheduled for elective surgery with curative intent for intraabdominal and intrathoracic cancer diagnoses (colorectal, thoracic, hepatobiliary or urologic) [4, 3]. The CFS is a feasible and accurate tool for assessing frailty [3] that has been used to screen participants with frailty into other prehabilitation trials [31]. A purposive sampling [32] technique was used in this nested qualitative study to identify participants in the PREHAB Study to obtain a distribution across inclusion criteria including age, sex and surgical speciality with the goal of gaining varied perspectives.

### Exercise prehabilitation

The intervention was a home-based total-body exercise training program based on a protocol with proven efficacy in improving the function of people without frailty in less than 4 weeks before surgery [33, 34]. All participants were exposed to at least 3 weeks of exercise, and participated for a mean of 5 weeks before surgery. Exercise prehabilitation consisted of three components: 1) strength training 2) aerobic exercise and 3) flexibility. Exercise prehabilitation was prescribed as 1-h sessions performed a minimum of 3 times per week. Participants were also provided with standardized nutritional advice. In addition to paper-based materials outlining the exercise prehabilitation program, participants received an individualized teaching session at the time of recruitment and were provided with a take home video, program guidebook, a pedometer and elastic exercise band. Activity logs and weekly phone calls were used to encourage and measure adherence and to answer questions. Adherence was defined as participation in at least 80% of the exercises included in the program [26]. The exercise program is described in detail elsewhere [18].

### Data collection

The interview guide was semi-structured and contained open-ended questions informed by the TDF (Additional File 2). Standard prompts were available to the interviewers (KD, CS) when needed.

Participants were contacted during their final week in the exercise prehabilitation program (i.e., the week before surgery) and were invited to participate in an interview at a time convenient to them. Interviews were conducted by trained clinical research assistants (KD, CS). Interviews were conducted by telephone, were digitally recorded and transcribed verbatim. The interviewers verified the content for accuracy. Any information that could potentially identify the participant was removed from the transcripts. The interviews ranged in length between 4 and 10 min. Transcripts were uploaded to and analyzed using NVivo 11 Software.

### Sample size

Our sample size was based on information power, a concept related to a narrow study aim, dense sample, and high quality and structured interviews, ultimately providing new knowledge that is aligned with the study aims [35]. We achieved information power after conducting 12 interviews as the research team collectively decided that the interviews provided high quality content and strongly supported the research question. An additional 3 interviews were conducted to check if any new information might be generated while providing additional data to support the existing codes and themes.

### Data analysis

The analysis followed a 3-step iterative approach: (1) coding, (2) generation of specific beliefs, and (3) generation of specific themes. Analysis and interviews occurred concurrently. First, transcripts were coded independently by two research assistants using conventional deductive content analysis [36]. The coders met weekly to compare their coding and to reach consensus on the TDF domains (as proposed in the interview guide) corresponding to each code generated. A third coder was involved to resolve disagreements (DIM). Next, coders generated specific beliefs, which refers to a collection of participant responses with a similar underlying theme that suggests a problem or influence on the target behaviour. Each code was rewritten as a specific belief by an individual team member (KD) and verified by the second team member (CS). A third coder was involved to resolve disagreements (DIM). Finally, similar specific belief statements were grouped to form common themes (KB, KD, EH, DIM). Themes represented a higher-level categorization of the data, with each theme subsuming multiple belief statements of similar nature. We used NVivo software 9.0 (QSR International) to code the analyses.

### Rigor

We worked as a team to ensure rigor was maintained throughout the course of this study. We kept thorough record of coding by two coders and the decisions made to reach consensus which provides an audit trail including notes made by the research team. Importantly, team members engaged in reflexive dialogue throughout data collection and analysis [37, 38].

## Results

### Participants

Fifteen participants (8 female; 7 male) from The PREHAB Study consented to participate in the semi-structured interviews. The average age was 72 years; the median CFS score was 4; the average number of weeks in the exercise prehabilitation program was 5. Within the 6 months leading up to surgery, two participants received neoadjuvant chemotherapy and one participant received neoadjuvant radiation therapy. See Table 1 for participant characteristics including prehabilitation adherence. Among the 3 participants who received preoperative neoadjuvant therapy, they were all at least 85% adherent to the program.

**Table 1:** Participant characteristics

Mean age (range)	72 (60–85)
Female, n (%)	8 (53)
Male, n (%)	7 (47)
Surgical Specialty, n (%)	
Colorectal	6 (40)
Thoracic	6 (40)
Hepatobiliary	3 (20)
Median CFS score (range)	4 (4–6)
Procedure Type, n (%)	
MIS	7 (47)
Open	8 (53)
Mean weeks in prehab (range)	5 (2–9)
Adherence, n (%)	
< 50%	3 (20)
75–79%	2 (13)
80–84%	5 (34)
85–89%	3 (20)
> 90%	2 (13)

*CFS* Clinical Frailty Scale, *MIS* minimally invasive surgery

### Themes

Nine key themes were identified, and aligned with 10 of the 14 domains of the TDF (relevant TDF domains denoted in parentheses): 1) Pre-existing conditions, fatigue and baseline fitness (*Beliefs about capabilities*); 2) Weather (*Environment*); 3) Guilt and frustration when unable to exercise (*Emotion, Reinforcement*); 4) The program being manageable and well-suited for older adults with frailty (*Beliefs about capabilities, Environment, Skills, Goals*); 5) Adequate resources to support engagement with the program (*Environment*); 6) Support from others helps with self-perceived adherence (*Social influences*); 7) A sense of control, intrinsic value, noticing progress and improving health outcomes (*Beliefs about consequences, Social professional role, Reinforcement*); 8) Enjoyable and facilitated by previous experiences (*Reinforcement, Skills*); 9) A need for individualization and variety (*Behavioural regulation*). Key themes and belief statements acting as barriers are presented in Table 2 and facilitators in Table 3; the number and proportion of respondents whose responses were mapped to each theme are also reported. Additional File 3 provides a full set of supportive quotes for each belief statement, grouped by theme.

**Table 2:** Barriers to adherence to exercise prehabilitation

Theme	Belief Statements	TDF Domain	Total	(%)
**Pre-existing conditions, fatigue and baseline fitness**	Pre-existing conditions, feeling tired and my initial state of fitness made the program difficult to complete	Beliefs about Capabilities	11	73%
**Weather**	The weather impacted my ability to do the prehab program	Environment	6	40%
**Guilt and frustration when unable to exercise**	I felt guilty when I did not complete the prehab exercises	Emotion, Reinforcement	4	27%
	I felt frustrated when I could not complete some of the prehab exercises	Emotion	4	27%
**A need for individualization and variety**	A more individualized program would be helpful to improve the prehab program	Behavioural Regulation	4	27%
	More equipment (i.e. resistance bands at different levels) would improve the prehab program	Behavioural Regulation	4	27%

**Table 3:** Facilitators to adherence to exercise prehabilitation

Theme	Belief Statements	TDF Domain	Total	(%)
**The program is manageable and well-suited for older adults with frailty**	The program is easy	Beliefs about Capabilities, Environment	15	100%
	There are no specific skills required to complete the prehab program	Skills	13	87%
	The prehab program is well-suited for me	Environment	10	67%
	The program had attainable goals	Goals	5	33%
**There are adequate resources to support engagement with the program**	The materials (program booklet and video) and weekly calls helped me complete the exercise program	Environment	9	60%
**Support from others helps with self-perceived adherence**	My family and friend's support helped me complete the prehab program	Social Influences	13	87%
	Exercising with someone else helped with completing the prehab exercise	Social Influences	5	33%
**A sense of control, intrinsic value, noticing progress and improving health outcomes**	I am able to have an impact on my own care or outcomes as doing the prehab program will have benefits for me down the road	Beliefs about Consequences, Social Professional Role	11	73%
	I am motivated to do the exercises	Goals	6	40%
	Seeing progress and improvement helped me to complete the prehab program	Reinforcement	5	33%
**Enjoyable and facilitated by previous experiences**	I enjoyed the prehab program	Reinforcement	14	93%
	Having experience with exercise in past helped me complete the prehab program	Skills, Reinforcement	6	40%
**A need for individualization and variety**	A more individualized program would be helpful to improve the prehab program	Behavioural Regulation	4	27%
	More equipment (i.e. resistance bands at different levels) would improve the prehab program	Behavioural Regulation	4	27%

#### Theme 1: Pre-existing conditions, fatigue and baseline fitness

Eleven respondents expressed that pre-existing health status and related symptoms impacted their ability to participate in prescribed exercise sessions. In all instances, this theme was identified as a barrier to participation. Low back and other chronic musculoskeletal conditions were often identified as influencing participants’ ability to exercise (e.g., ‘I think there were a couple of the exercises that I didn’t do because of my lower back issues’). Some participants expressed that these physical limitations reduced the amount of time that they could spend exercising, while for others, limitations were related to the specific exercises they could perform.

A feeling of tiredness was also commonly identified as a pre-existing barrier. For some participants prevalent tiredness impacted their ability to exercise on a given day (e.g., ‘There’s a degree of tired where I just can’t do it. So, it was always on those times I just didn’t have it in me.’), while for others fatigue set in while exercising (e.g., ‘I was very tired after I got done with them and it said do them 10 times. I couldn’t do the 10 times.’).

For some participants, existing health status presented a challenge when starting the program (e.g., ‘Well, when this all first started, it was very hard to do.’). However, as the program advanced, some participants found it easier to participate (e.g., ‘I wasn’t used to exercising. So, yes, I’d go out and take a walk, but I mean I wasn’t walking like I’m walking now. Now, I do it like eight times a day, where before I only went to the gate and maybe back again.’, and ‘I worked my way kind of up to what I expected I should do. But I found it very hard when I started at first.’).

#### Theme 2: Weather

Adverse weather conditions were identified as a barrier to participation, particularly for the aerobic component of the program. In some cases, heat and humidity impacted participant’s motivation to exercise (e.g., ‘…on those really hot humid days. You just didn’t feel like doing [the exercises].’). Cold and winter weather were also identified as barriers (e.g., ‘It’s been too cold to walk outside. Especially when I hate the cold to begin with.’, and ‘It would have been easier to schedule the walks for sure had the weather been nice.’).

#### Theme 3: Guilt and frustration when unable to exercise

We identified belief statements related to guilt and frustration from participants when they were unable to exercise. In each case this was identified as a barrier as participants did not frame their experience as motivating; negative emotions were expressed (e.g., ‘I thought I should have gone out [walking], but I didn’t. But I did, I felt guilty.’, and ‘I was always frustrated because oh, come on I’d tell myself, you can do this. But my body was saying I don’t think so. So that’s different for me. I’m always used to pushing, so I didn’t feel very good about it when I didn’t do it.’).

#### Theme 4: The program is manageable and well-suited for older adults with frailty

All participants believed the program was easy to follow, which was identified as an important facilitator (e.g., ‘I found it fairly easy, but probably hard enough for my age.’), while environmental aspects, such as the home-based structure of the program, further facilitated participation (e.g., ‘I think, to me, it was easier to do it at home and do it at your own time and that, as opposed to doing it in a group that you have to get there and do it as a group then come back. At least at home here, with my wife, I can do it at my own pace and my own time.’).

Participants also consistently expressed beliefs that a lack of exercise related skills did not act as a barrier to participation (e.g., ‘It was something that everybody should be able to do.’). Teaching and support provided via multimodal instructions helped to guide successful participation in the program (e.g., ‘It wasn’t particularly any skills, just as long as you can watch the video and do what you have to do at the pace you can do it, that’s all.’, and ‘Originally, I didn’t quite understand what was said in the book but the video that you had me have a look at, all I had to do was read it once and it took away any uncertainties I had about what they wanted.’). The program was also felt to be well suited to the needs and capabilities of participants, proving a challenge but not being overly difficult to de-motivate (e.g., ‘They just weren’t super strenuous exercises. They were just moderate exercises… But they were good. They weren’t too hard that wanted to make you quit.’, and ‘Yeah, not asking me to do anything that was impossible or disheartening because I couldn’t do it, you know?). Finally, attainable goals appeared to facilitate participation (e.g., ‘[the goals of the program] were well within my capacity.’, and ‘And then at least this was realistic. Like, it was easier and not feeling like you were constantly failing.’).

#### Theme 5: There are adequate resources to support engagement with the program

Program materials and structures were identified as facilitators to participation. Participants expressed that provision of written and audiovisual guides helped them to participate (e.g., ‘It’s easy to follow, no problem, especially with the video and that and the book. And it’s laid out very good, so you can follow it, no problem.’, and ‘Yes, because I was able to download the media file, so I was able to watch it on my TV, or I have it on my phone so I can have it with me so I can do some exercise even if [my wife] wasn’t around. Access to the file is very helpful.’). It was also identified that follow up provided via telephone during the program supported participants in successfully exercising (e.g., ‘Yeah, there was a girl named Chelsey who called me every week to check on my progress. Always enjoyed talking to her and it showed me that you guys have me doing this, but you’re also really interested in how I’m doing and that I thought was a good thing. That you kept up your end as I was doing mine. I thought that was very good.’, and ‘I think if you had to turned me lose with the book and a TheraBand and a walker and not checked up on me every week, I’m not saying I would have bowed out, but I think there would be a tendency for others to lose interest and say look, I’m not doing this.’).

#### Theme 6: Support from others helps with self-perceived adherence

Participants identified the importance of social influence via support from family and friends as important facilitators to participation (e.g., ‘Now, [my partner] wants me to do the exercises so I’m ready for the operation and that, as opposed to if I was alone and doing them alone. Maybe I would have not done them as much as I did.’, and ‘Well, I have support from my whole family… because there are times you don’t feel like doing it and they just—oh you’ve got to get it done.’). Furthermore, completing exercises with another person also supported completion of prescribed exercise (e.g., ‘Doing it with another person makes it a lot easier so that you can go through the exercise properly.’, and ‘Well my husband walks with me all the time. And I never walked alone, he always walked with me.’).

#### Theme 7: A sense of control, intrinsic value, noticing progress and improving health outcomes

Participants identified thinking that participating in prehabilitation helped them to feel like they were impacting their own care in a way that would benefit them in the longer term. These beliefs appeared to facilitate participation through influence on participant’s beliefs about consequences of their exercising (e.g., ‘Well I really want to do well in the surgery, as well as I can. So, it gave me something to do, like something concrete I could do to try and make it better… because in some ways there’s not too much I can do but that was one thing I could do… to be as strong for the surgery as I could be.’) and their role in impacting their health outcomes (e.g., ‘Well I think I felt good because I felt I was doing something to help myself through the surgery. I felt good about that.’). Participating in exercise was also identified as motivating participants toward their goal of achieving a positive surgical outcome (e.g., ‘I thought it was probably very positive to do and hopefully I see benefits down the road sometime’, and ‘I think just the fact that I’m anxious to have the surgery and get it over with, and I’m hoping that these exercises are going to make [surgery] easier for me’.)

Successful completion of exercises and a sense of improvement in performance over time also appeared to facilitate participation (e.g., ‘As you progress during the weeks and that, right now I feel great and I love doing them. And they get easier as you go along.’, and ‘I feel a bit better because I had not been exercising, so I can tell my breathing is better. I feel stronger. So I feel more physically ready than I did five weeks ago or six weeks ago.’).

#### Theme 8: Enjoyable and facilitated by previous experiences

Enjoying participation in the prehabilitation program appeared to reinforce and facilitate adherence to exercise before surgery (e.g., ‘No, like I said, I feel great after. It’s a sense of accomplishment. So, I’m one step closer to the operation and one step more prepared for the operation.’, and ‘I found I felt better after doing cardio, as opposed to when I didn’t. It is good to do, for sure. Yeah, no it all felt good to do, for sure.’). Furthermore, having past experience with exercise appeared to further facilitate participation (e.g., ‘Well, you have to have a little bit of strength because I noticed with my sister some of the exercises I had been doing, I could do 20, and she had to stop after five or something like that.’, and ‘I think that was pretty good. The chair squats and that were some of the things I did before. And the calf raises and stuff like that I’ve done before.’).

#### Theme 9: A need for individualization and variety

This theme was identified as both a barrier and a potential facilitator and is therefore included in both Tables 2 and 3. Participants identified opportunities to individualize the exercise program as a potential facilitator to participation. Some participants identified personalization of specific exercises to their preferences and abilities as important (e.g., ‘I think to go through the exercises that are available… making it clear that if you can’t do it, don’t do it. If you go through each one to determine which is best suited to the patient, and then they can focus on those ones and not be thinking about the ones that they can’t do.’). Surgery-specific personalization was also highlighted (e.g., ‘I mean, because I’m having part of my lung removed, you know, they’ve given me some deep breathing… I guess depending on what surgery people are having, it might be more specific to a certain surgery to do certain things.’).A lack of variety in exercises was identified as a barrier to participation (e.g., ‘But once you’re just repeating there’s nothing new, I find it gets a bit not as interesting.’). Greater access to resistance bands of different difficulty levels was requested by several participants, highlighting that access to equipment could act as a barrier to exercise (e.g., ‘I’m just wondering in the bands you gave us, like I have the rubber ones I had way back when, do they have different strengths or different resistance to those bands.’).

## Discussion

In this qualitative study of older patients with frailty participating in a home-based exercise prehabilitation program prior to cancer surgery, we identified patient-reported barriers and facilitators of adherence to exercise that aligned with 10 of the 14 domains of the TDF. As adequate adherence is likely a key mediator of prehabilitation efficacy, future program design should consider approaches that leverage facilitators and overcome barriers that we have identified. Key facilitators appear to include that the program was manageable and suitable to older adults with frailty, there were adequate resources to support engagement, and participants felt a sense of control over their health and their health outcomes. Furthermore, older adults with frailty expressed no need for previous experience, and perceived better adherence when social support from family or an exercise partner were available. Successful programs will need to identify means to overcome barriers such as pre-existing fatigue and physical ailments, impacts of adverse weather conditions, and emotional support when feelings of guilt or frustration occur where sessions are missed. A need for individualization and variety within the program was offered as a suggestion by participants and was therefore described as both a facilitator and a barrier. Future studies should explore using a tailored approach to prehabilitation targeting individual needs of participants. Individualization could include providing different levels of exercises and equipment, modifying the program based on limitations and skills and exploring exercise options using an interests-based approach.

Adherence to prescribed exercise is consistently identified as a challenge in studies aimed at leveraging physical activity to improve health [39, 40]. Prior to surgery, prehabilitation has emerged as a high priority intervention to enhance outcomes and exercise interventions are included in over 80% of prehabilitation programs [15]. However, the average adherence to prehabilitation programs is only 70% [15], and appears to be even lower in prehabilitation programs specifically tested in older people with frailty [17, 18]. In the two available non-pilot prehabilitation trials specific to older people with frailty, mean adherence has been ~ 60% and intention to treat efficacy analyses (i.e., those that include all participants randomized to prehabilitation regardless of adherence) have shown no strong evidence of prehabilitation improving postoperative function or complication rates. However, when analyses have been limited to participants completing at least 80% of their exercises, both available trials suggest substantial decreases in complication rates and improvement in functional recovery with prehabilitation. Therefore, understanding how to optimize adherence appears to be a key requirement to deliver effective prehabilitation programs for people with frailty.

Although initially developed to guide implementation science related to health professionals’ behaviour, the TDF has since been used to assess barriers and facilitators to behavior change in a variety of settings, including for patients [41]. While quantitative assessments of predictors of adherence to exercise can help to identify patient and program characteristics that are associated with adherence [42], qualitative research provides a complimentary approach where participants can identify and explore factors that they perceive as relevant, which can then be mapped to evidence-based strategies to leverage facilitators and overcome barriers [43]. Triangulation between quantitative and qualitative findings can also be used to develop a deeper understanding of the multiple factors that may lead to optimal design of prehabilitation programs.

Previous qualitative assessment of patients’ perspectives regarding prehabilitation have not focused on patients with frailty, but highlight several findings consistent with barriers and facilitators identified in the current study [44, 45]. Similar to Ferreira and colleagues (who studied patients without frailty enrolled in a hospital-based prehabilitation program), as well as Gillis and colleagues (who studied the views on prehabilitation from patients participating in an Enhanced Recovery After Surgery (ERAS) program), participants in our study expressed enjoyment with the prehabilitation program and that the program was well-suited to their needs. Participants in all three studies also identified intrinsic value and motivation in seeing the results of prehabilitation improve their health before surgery. In contrast, participants in the current, home-based program identified the structure of the program as a facilitator, whereas for Ferreira and colleagues, transportation and parking were identified as the main barriers to participation in their hospital-based program.

The literature regarding adherence with exercise therapy in older people with frailty is sparse. Available reviews have either simply reported adherence rates [46], focused on the structural aspects of exercise programs [47], reported the need to address behavior change techniques due to limited data [48], or addressed limited indications for exercise [49]. By focusing on people with frailty preparing for cancer surgery, we identified several novel and important barriers. As individuals with frailty are in a state of vulnerable health by definition, it is crucial to recognize that aspects of baseline health and wellness can act as important barriers to adherence. This means that for people with frailty, programs more tailored to their limitations, day to day energy levels, and symptom management may be required. In the current study, 3 participants were receiving either preoperative chemotherapy or radiation and were able to adhere (≥ 85%) to the program. While these neoadjuvant treatments can be very challenging physically and emotionally, the results of this study suggest that ongoing support and encouragement from prehab coaches may facilitate adherence. It is also a testament to the resiliency and motivation of older adults with frailty preparing for cancer surgery. Our results further suggest that adherence strategies may need to account for seasonal and geographic considerations, as both summer and winter weather were identified as barriers to aerobic components of the program. Finally, while home-based programs have been identified as a priority for people with frailty [19], and a facilitator in the current study, virtual outreach and support will need to be optimized to provide access to services such as emotional support and motivational interviewing, as negative emotions like guilt and frustration were clearly identified as barriers to adherence by our participants. It is likely that effective partnerships with patients, as highlighted by Gillis and colleagues, will be required in parallel with evidence-based design of study processes aligned with TDF tools such as the Behaviour Change Wheel or Matrix [43, 50].

### Strengths and limitations

This study should be considered in context of its strengths and limitations. As a qualitative study, results could be influenced by a variety of factors, including intrinsic biases of interviewers, coders and researchers. While we used best practices to recruit our sample and to achieve data adequacy, there may be selection bias as our participants were individuals willing to both participate in a randomized trial of prehabilitation and a nested, qualitative study. The current study focuses on people living with frailty who required oncologic surgery. The duration of prehabilitation was generally in line with other programs described in the literature [12, 18, 35]. The findings, however, may not generalize to other surgical settings where wait times are longer (and therefore time for prehabilitation longer), such as non-oncologic thoraco-abdominal-pelvic surgeries, or orthopedic procedures such as joint replacement. While using the TDF allowed us to apply a robust and well-studied behaviour change framework, alternative approaches to qualitative evaluation might be expected to uncover different phenomena. Further, adherence rates for most participants (12/15) in this study was > 75%. Future work should expand on the findings of this study to further explore the experiences of people with lower levels of adherence to exercise interventions, so that their unique considerations could be adequately represented. Finally, future assessments of barriers and facilitators that come from a sample of respondents drawn from multiple jurisdictions would also be useful to complement our single center study.

## Conclusions

For older people with frailty participating in exercise prehabilitation before cancer surgery, many facilitators to program adherence align with those of patients without frailty, including the intrinsic motivation of improving one’s health before surgery, having social support and the use of purpose designed and personalized programming. Future programs should leverage these facilitators while considering novel barriers such as limitations due to poor baseline health, adverse weather conditions, and need for emotional support. Continued, iterative implementation and re-evaluation of barriers and facilitators may be required to ensure older people with frailty achieve adequate adherence to benefit from prehabilitation.

## Supplementary Information

**Additional file 1.** COREQ (COnsolidated criteria for REporting Qualitative research) Checklist.

**Additional file 2.** Interview Guide.

**Additional file 3.** Supportive Quotes for Each Belief Statement, Grouped by Theme.

## Data Availability

All data generated or analysed during this study are included in this published article [and its supplementary information files]. Any additional data are available from the corresponding author on reasonable request.

## Abbreviations

CFS: Clinical Frailty Scale
ERAS: Enhanced Recovery After Surgery
TOH: The Ottawa Hospital
TDF: Theoretical Domains Framework
