# Supplementary material for: Barriers and facilitators to participation in exercise prehabilitation before cancer surgery for older adults with frailty: a qualitative study
Source: BMC Geriatr. 2023 Jun 6;23:356. doi: 10.1186/s12877-023-03990-3 (PMC10242997; doi:10.1186/s12877-023-03990-3)
Supplement: Supplementary file 2 — Additional file 2. Interview Guide. [file 12877_2023_3990_MOESM2_ESM.docx]

Prehab – Interview Guide

1. How easy or difficult did you find the prehabilitation program?
   - 1. Prompts:
        1. What about the program made it easy?
        2. What about the program made it difficult?
2. Do you think there any specific skills required to participate in the prehabilitation program? If so, what?
3. Was there anything in your home environment that helped or hindered your participation in the prehabilitation program?
4. Did you have any support from family/friends while doing the prehabilitation program? If so, can you speak about any support from family/friends that you had while completing the prehabilitation program?
5. Were there any factors or situations helped you in completing the prehabilitation program? Were there any factors that hindered you from being able to complete it?
   1. What were the factors?
   2. Prompts:
      1. Something in your home? Was there anything missing to be able to do the prehabilitation program at home?
      2. In the environment?
      3. People or family? Were they supportive or unsupportive?
6. How did you feel when doing the prehabilitation program (when you were able to complete a session?)
7. How did it feel if you were unable to complete a session (i.e., if you were unable to do your cardio sessions one week)?
8. Do you think there is anything that could be done to improve the prehabilitation program?
   1. Prompt: Is there anything that would have helped you in the process that you did not have and would have liked? Any other resources?
