# Supplementary material for: Barriers and facilitators to participation in exercise prehabilitation before cancer surgery for older adults with frailty: a qualitative study
Source: BMC Geriatr. 2023 Jun 6;23:356. doi: 10.1186/s12877-023-03990-3 (PMC10242997; doi:10.1186/s12877-023-03990-3)
Supplement: Supplementary file 3 — Additional file 3. Supportive Quotes for Each Belief Statement, Grouped by Theme. [file 12877_2023_3990_MOESM3_ESM.docx]

**Additional File 3**

**Supportive Quotes for Each Belief Statement, Grouped by Theme**

| **Theme** | **Belief Statements** | **Quotes** |
| --- | --- | --- |
| **Pre-existing conditions, fatigue and baseline fitness** | | |
|  | Pre-existing conditions, feeling tired and my initial state of fitness made the program difficult to complete. | “*There’s a degree of tired where I can push myself and do stuff, but there’s a degree of tired where I just can’t do it. So, it was always on those times I just didn’t have it in me.”* |
|  |  | “*Well, it was just that I never did it before I guess, and I wasn’t used to exercising. So, yes, I’d go out and take a walk, but I mean I wasn’t walking like I’m walking now. Now, I do it like eight times a day, where before I only went to the gate and maybe back again*.” |
|  |  | “*Well, when this all first started, it was very hard to do. I worked my way kind of up to what I expected I should do. But I found it very hard when I started at first.*” |
|  |  | "*Yeah, I wish I had done more but because of my back, it’s a real pain in the butt, because half an hour seems like my limit. Like, I can’t walk much more than maybe another five minutes outside, but it’s really interesting because I do this walk and like the last five minutes it’s like okay, it’s time to stop*." |
|  |  | "*Otherwise, I think there were a couple of the exercises that I didn’t do because of my lower back issues. So just the stand-to-sit, the squatting, that one, I have sciatica going on so I think there was that one and another one that I found I was getting pain with so I didn’t do*." |
|  |  | "*Yeah, my health. My hemoglobin levels didn’t allow me to do the cardiovascular I would have liked to have been able to do*." |
|  |  | "*Tired. I was very tired after I got done with them and it said do them 10 times. I couldn’t do the 10 times. I did about five, but I didn’t do all what they said to do.*" |
|  |  | "*The only one I didn’t do was the one that you sit on the chair and try to get up on your own. I just couldn’t do it. I even used the pillows on the couch and everything like that and it didn’t work*." |
|  |  | "*Actually, I was so tired. I had no feeling when I got home from Ottawa, like it was a big day. Instead of going two days, I did it all in one day and it was a lot. It was a lot*." |
|  |  | "*Yes, I had to go and have a stent placed and that day I didn’t do it. And the day after, I wouldn’t say I felt sick exactly, but I just felt a bit discombobulated if that’s not too big a word, so I took a day off that day as well. But apart from those two days, the first day when I had the stent placed I had to be down to the hospital fairly early and then when I got home I didn’t feel like going out. But I had my reasons.*” |
|  |  | "*Mobility issues were the only concerns I had. Yes, I have a tear in my right shoulder and in my lower back I’ve got two compressed discs and a left knee.*" |
|  |  | "*No, I didn’t, but I didn’t do them very well for a couple of weeks because of my neck*." |
| **Weather** | | |
|  | The weather impacted my ability to do the prehab program. | *“…some days it was on those really hot humid days. You just didn’t feel like doing [the exercises]*.” |
|  |  | “*There were a couple of rainy days that I only went out to the garage and went around the car and walked in the house … so I didn’t have too many steps on those rainy days*.” |
|  |  | “*It’s been to cold to walk outside. Especially when I hate the cold to begin with.*” |
|  |  | "*Yeah, for me I think just because so many visitors prior to surgery and family things and whatever. So yeah, it was not always easy. It would have been easier to schedule the walks for sure had the weather been nice*." |
|  |  | "*I couldn’t go outside some of those hot days. I just did the exercises inside. But I got up at six and I ran then.*" |
| **Guilt and frustration when unable to exercise** | | |
|  | I felt guilty when I did not complete the prehab exercises. | “*[on the days I couldn’t exercise] I felt like something was missing. I felt a little guilty, I guess*.” |
|  |  | “*Well, you know, I did, especially the day after. I thought I should have gone out [walking], but I didn’t. But I did, I felt guilty*.” |
|  | I felt frustrated when I could not complete some of the prehab exercises. | “*There was that one day that I couldn’t do what I wanted to do and that was on the touch squats… my hip was so sore all I could grind out was four of them… [I was] sore and disappointed that I couldn’t do what I should have been able to do*.” |
|  |  | “*I was always frustrated because oh, come on I’d tell myself, you can do this. But my body was saying I don’t think so. So that’s different for me. I’m always used to pushing, so I didn’t feel very good about it when I didn’t do it*.” |
|  |  | “*It’s kind of depressing. It gets frustrating and you want to do it, but you can’t. But if it’s explained to you why you shouldn’t do it and then focus on the ones that the patient can do, they’ll be more apt to instead of decreasing the amount of those one [they can’t do]*.” |
|  |  | "*I didn’t skip. I just didn’t do it as well. It was frustrating*." |
| **A need for individualization and variety** | | |
|  | A more individualized program would be helpful to improve the prehab program. | “*I guess depending on what surgery people are having, it might be more specific to a certain surgery to do certain things*” |
|  |  | “*I think to go through the exercises that are available, to go through it like when you’re doing the intake with the patient, to go through the exercises to determine which ones are more suited to them. And making it clear that if you can’t do it, don’t do it. For a follow up perspective, because let’s say there’s 10 exercises, if you go through each one to determine which is best suited to the patient, and then they can focus on those ones and not be thinking about the ones that they can’t do*.” |
|  |  | "*No, I didn’t find it difficult. As I said, that kind of lost something for the exercises but that’s me. I tend to kind of get tired of them at the beginning is good because I was learning how to do them and stuff. But once you’re just repeating there’s nothing new, I find it gets a bit not as interesting. I never found them difficult*." |
|  |  | "*I’m not sure. I mean, I guess the only thing, I guess, would be specific to a person’s surgery. I mean, because I’m having part of my lung removed, you know, they’ve given me some deep breathing, which I had been doing some yoga with diaphragmatic breathing, but that’s important before a lung surgery anyway. So, I guess depending on what surgery people are having, it might be more specific to a certain surgery to do certain things. But I guess that’s hard to do. I mean, any cardio, any fitness, any strength training is good. And I guess for my age group, I guess keeping it simple is probably the best way to manage it and keeping it standardized. I guess that is best, for sure*." |
|  | More equipment (i.e. resistance bands at different levels) would improve the prehab program | “*an option between a band and the yellow one that’s provided, at the intake could basically see which one is better suited to them*.” |
|  |  | "*The only thing I thought of was maybe I should have been listening to music or something, like aerobic music or something to get me doing them*." |
|  |  | “*I don’t think so really because all of it is pretty easy. Maybe you should add more things or little weights…Like you know with the arm curls and stuff like that with weights. When I first ever started, well I only do two pound ones, but they started me at one pound ones and they weren’t heavy enough at one pound. But for some people one pound would be a lot, like that sort of thing*.” |
|  |  | "*No, not really. I’m just wondering in the bands you gave us, like I have the rubber ones I had way back when, do they have different strengths or different resistance to those bands*?" |
|  |  | "*The only thing I didn’t have is the bands are very loose. We modified how we hold them, but if you had an option between a band and the yellow one that’s provided, at the intake could basically see which one is better suited to them*." |
| **The program is manageable and well-suited for older adults with frailty** | | |
|  | The program is easy. | "*I didn’t have any difficulties with any of it*.” |
|  |  | “*No, I think, to me, it was easier to do it at home and do it at your own time and that, as opposed to doing it in a group that you have to get there and do it as a group then come back. At least at home here, with my wife, I can do it at my own pace and my own time*.” |
|  |  | "*I found it fairly easy, but probably hard enough for my age*." |
|  |  | "*I’d give it a three. It wasn’t really easy, but it wasn’t all that hard to do either*." |
|  |  | "*Well, I guess the ease with which I did the stipulated exercises and they fulfilled the time allocation*." |
|  |  | "*It’s easy to follow, no problem, especially with the video and that and the book. And it’s laid out very good, so you can follow it, no problem*." |
|  |  | "*I found it easy to follow, but you have to take into consideration a person’s limitations.*" |
|  |  | "*They just weren’t super strenuous exercises. They were just moderate exercises I would say, you know? But they were good. They weren’t too hard that wanted to make you quit*." |
|  | There are no specific skills required to complete the prehab program. | “*It was something that everybody should be able to do*” |
|  |  | “*It wasn’t particularly any skills, just as long as you can watch the video and do what you have to do at the pace you can do it, that’s all*.” |
|  |  | “*Not really skills, you’ve just got to be determined that you’re going to do it, I guess*.” |
|  |  | *“Well, I think anybody can do it.”* |
|  |  | “*I was very comfortable doing it. I didn’t have any concerns. Basically as explained to me: do what I can, don’t [over] exert, and basically don’t injure myself or trying to attempt something that I know that I’m not comfortable in doing*.” |
|  |  | "*Yes. Originally, I didn’t quite understand what was said in the book but the video that you had me have a look at, all I had to do was read it once and it took away any uncertainties I had about what they wanted*." |
|  |  | "*Yeah, you’d have to be able to read, other than that, no. These are things that not everybody can do, but after you’re done the program you can do them better than you could when you started. And I noticed that, particularly with the stretching ones. I found them onerous for the first while. But even though I didn’t like doing them at the end, they were easier to do*." |
|  |  | *“I’m thinking well if you’re younger than that then it wouldn’t—I mean, you could do a lot more. But no, I think it’s quite reasonable over 60.”* |
|  |  | "*But I think they’re pretty straightforward exercises, so should be fine for most people*." |
|  | The prehab program is well-suited for me. | “*They just weren’t super strenuous exercises. They were just moderate exercises… But they were good. They weren’t too hard that wanted to make you quit*.” |
|  |  | "*I think it’s well organized and I think you do a great job at follow up and I think that’s important. I think if you had to turned me lose with the book and a TheraBand and a walker and not checked up on me every week, I’m not saying I would have bowed out, but I think there would be a tendency for others to lose interest and say look, I’m not doing this. So kudos to you*." |
|  |  | "*Yeah. Yeah, not asking me to do anything that was impossible or disheartening because I couldn’t do it, you know*?" |
|  |  | "*Well probably if someone was in better shape than me it might be easy, but for me it wasn’t too easy*." |
|  |  | "*I don’t think a whole lot. As long as you don’t push people too hard with hard exercises. No, I think you’ll be fine. I think it’s a good star*t." |
|  |  | "*It is, yes. Yes, some of the stretching ones, they keep you going. Do you know what I mean? After you’ve done it, you know you’ve been there*." |
|  |  | "*No, that part yes sometimes, because, you know, just because of life but besides that, no. I mean, it’s all things, you know, swimming and golfing and walking, and it’s all things that I love doing*." |
|  | The program had attainable goals. | “*[the goals of the program] were well within my capacity*.” |
|  |  | “*They just weren’t super strenuous exercises. They were just moderate exercises I would say, you know? But they were good. They weren’t too hard that wanted to make you quit*.” |
|  |  | “*I think it’s good there’s just a few weeks, like four weeks or something because I think if it was going to be six or seven weeks I might not have kept up with the exercises*.” |
|  |  | "*Well, it is. And we take them in the forest, so you’ve got to get in the car and you’ve got to go there and get—so, I mean even though it’s 30 minutes, it is more like 45, 50 minutes to do it. So when they said that, I went not gonna happen. But anyway, so that was not realistic. And then at least this was realistic. Like, it was easier and not feeling like you were constantly failing.*" |
| **There are adequate resources to support engagement with the program** | | |
|  | The materials (program booklet and video) and weekly calls helped me complete the exercise program | “*…the video that you had me have a look at, all I had to do was read it once and it took away any uncertainties I had about what they wanted*.” |
|  |  | “*It’s easy to follow, no problem, especially with the video and that and the book. And it’s laid out very good, so you can follow it, no problem*.” |
|  |  | “*Yes, because I was able to download the media file, so I was able to watch it on my TV, or I have it on my phone so I can have it with me so I can do some exercise even if [my wife] wasn’t around. Access to the file is very helpful*.” |
|  |  | "*That’s fair, yes. But at the same time, I was glad to see the video*" |
|  |  | "*Yes. Originally, I didn’t quite understand what was said in the book but the video that you had me have a look at, all I had to do was read it once and it took away any uncertainties I had about what they wanted*." |
|  |  | "*The video and the pictures, yeah. I think that was fine*." |
|  |  | "*No, I don’t think you need background at all. Maybe with the stretching, but the video does it, though. So yeah, some of them I wasn’t sure but the video was quite clear. So no, I don’t think so. I think if you’re totally out of shape—I wasn’t—it might be more difficult*." |
|  |  | “*Yeah, there was a girl named Chelsea who called me every week to check on my progress. Always enjoyed talking to her and it showed me that you guys have me doing this, but you’re also really interested in how I’m doing and that I thought was a good thing. That you kept up your end as I was doing mine. I thought that was very good*.” |
|  |  | *“Just the order in which you start. From the standing pushup, giving you the options to go to the pushup, it’s easy flowing.”* |
|  |  | “*Not really. I mean I think it’s well organized and I think you do a great job at follow up and I think that’s important. I think if you had to turned me lose with the book and a TheraBand and a walker and not checked up on me every week, I’m not saying I would have bowed out, but I think there would be a tendency for others to lose interest and say look, I’m not doing this. So kudos to you*.” |
|  |  | “*No, other than thanks to Chelsea for following up every week. I think it’s an excellent part of the program and I think your study and the program would fail without that kind of adjudication from your end. I think it would be very easy for people like me to lose interest. I was a long time doing these exercises before my surgery was scheduled and it would be easy to as I say fall off the wagon I would think if you didn’t follow up*.” |
| **Support from others helps with self-perceived adherence** | | |
|  | My family and friend's support helped me complete the prehab program. | “*Well I have support from my whole family… because there are times you don’t feel like doing it and they just—oh you’ve got to get it done*." |
|  |  | “*It helps in a way that like I said before, some days ah, I don’t feel like doing it and she’s after me, “Well, you know you have to do it. Let’s do it”, you know, let’s start. And then we started and then we finished the whole thing. So she’s mostly my—how would I say it—my driving force behind it*.” |
|  |  | “*Now, [my partner] wants me to do the exercises so I’m ready for the operation and that, as opposed to if I was alone and doing them alone. Maybe I would have not done them as much as I did*.” |
|  |  | "*No, my wife was quite surprised. She supported me*." |
|  |  | "*Absolutely. My wife—boy she’s my rock. She’s my anchor. I’ll tell you, I’m a lucky man*." |
|  |  | "*No, they were supportive. Yes, anybody I mentioned it to you, yes. They thought it was good, yes*." |
|  |  | "*Yeah. I told people about it and they all thought it was a good idea but nobody kind of said how is it going or anything like that because I don’t think they remembered it*." |
|  |  | "*Yeah, because there are times you don’t feel like doing it and they just—oh you’ve got to get it done. So yeah, you just get it done*." |
|  | Exercising with someone else helped with completing the prehab exercise. | “*Doing it with another person makes it a lot easier so that you can go through the exercise properly*” |
|  |  | "*Well my husband walks with me all the time. And I never walked alone, he always walked with me. And even last summer with all the walking we did, it was he and I that did all the walking but the exercise things I did on my own like that*." |
|  |  | "*Yeah, he was always walking with me because again, I’m having some issues. I was fine going on my own but he would usually come along, which was good for him, too*." |
|  |  | "*Well, when it started, my friend was just doing her exercise for a knee replacement, so we could kind of relate with the exercise thing, but I can’t think of anything specific at home*." |
|  |  | "*It helps in a way that like I said before, some days ah, I don’t feel like doing it and she’s after me, “Well, you know you have to do it. Let’s do it”, you know, let’s start. And then we started and then we finished the whole thing. So she’s mostly my—how would I say it—my driving force behind it*." |
| **A sense of control, intrinsic value, noticing progress and improving health outcomes** | | |
|  | I am able to have an impact on my own care or outcomes as doing the prehab program will have benefits for me down the road. | “*Well I really want to do well in the surgery, as well as I can. So it gave me something to do, like something concrete I could do to try and make it better… because in some ways there’s not too much I can do but that was one thing I could do… to be as strong for the surgery as I could be*.” |
|  |  | “*I mean I never find myself thinking this is a waste of time or this doesn’t make sense. I mean it was all relative and I knew I was benefitting from it*.” |
|  |  | "*I think just the fact that I’m anxious to have the surgery and get it over with, and I’m hoping that these exercises are going to make it easier for me*." |
|  |  | "*Well I think I felt good because I felt I was doing something to help myself through the surgery. I felt good about that*." |
|  |  | "*No, like I said, I feel great after. It’s a sense of accomplishment. So I’m one step closer to the operation and one step more prepared for the operation*." |
|  |  | "*Yeah, because you need your arms, some muscles to push yourself up sometimes. It all depends what kind of disease you have. I lost a lot of my muscle and everything when I started with the arthritis and the medications I’m on and I lost pretty well all my muscle and that then, so what little bit I have I’ve got to try to keep what I have*." |
|  |  | "*It didn’t bother me to be doing it at all and I knew there was a reason for it, to help my breathing and what muscles I have left*." |
|  |  | "*Well with any luck, it’ll put me in better shape when I have my surgery on Tuesday. I’m hoping*." |
|  |  | "*I feel a bit better because I had not been exercising, so I can tell my breathing is better. I feel stronger. So I feel more physically ready than I did five weeks ago or six weeks ago*." |
|  | I am motivated to do the exercises. | “*… just like I said determination on your own part. You have to have that*.” |
|  |  | “*I wanted to do it, because I felt I needed to do it. And I had to prepare myself for surgery and exercise prepared my mind too for it*.” |
|  |  | "*I felt that I got a workout every day that I did it because again, I didn’t do just the minimum. I did all that I could and some days all that I could wasn’t enough. There was one day, it was the chair squats I think that you had a look at that. I was only able to do four instead of my usual 12*." |
|  |  | "*Well I really want to do well in the surgery, as well as I can. So it gave me something to do, like something concrete I could do to try and make it better. So that was good. I like that part*." |
|  |  | "*For the running, I think it’s because I’ve just always been doing it and I think it’s not quite the same because I used to just kind of run in my head with all sorts of stuff, but because I can’t just get into it like I used to, it’s not quite as satisfying. But I still like it, so I just keep doing it. And my goal is for after I’m finished all my treatments, which won’t be for quite a while, I’m really looking forward to just getting up and doing it like I used to. I hope that works*." |
|  |  | "*Yeah, because you need your arms, some muscles to push yourself up sometimes. It all depends what kind of disease you have. I lost a lot of my muscle and everything when I started with the arthritis and the medications I’m on and I lost pretty well all my muscle and that then, so what little bit I have I’ve got to try to keep what I have*." |
|  |  | "*When you didn’t do it, you just keep it in the back of your mind that you’ve got to get it done. You’ve got to get it done. You have to have a little determination to be able to complete it because a lot of people don’t want to be bothered, because some of the exercises don’t seem like they make sense but I know they all do*." |
|  |  | *“Pretty well my own determination.”* |
|  |  | “*No, it’s just a determination to get it done. And you do feel better afterwards, for sure*.” |
|  |  | "*I don’t know. I get determined, I just do what I have to do and that’s it*." |
|  |  | "*The main motivation: feel better*." |
|  | Seeing progress and improvement helped me to complete the prehab program | “*As you progress during the weeks and that, right now I feel great and I love doing them. And they get easier as you go along*.” |
|  |  | “*I feel a bit better because I had not been exercising, so I can tell my breathing is better. I feel stronger. So I feel more physically ready than I did five weeks ago or six weeks ago*.” |
|  |  | "*These are things that not everybody can do, but after you’re done the program you can do them better than you could when you started. And I noticed that, particularly with the stretching ones. I found them onerous for the first while. But even though I didn’t like doing them at the end, they were easier to do*." |
|  |  | "*Yes, maybe I am. And my back wasn’t sore. So that was the biggest thing was I’d like to go longer. I’d like to do it longer but I couldn’t. And, you know, it’s like we went to the learning thing and they said 45 minutes a day, twice a day and it’s like that’s just not going to happen*." |
| **Enjoyable and facilitated by previous experiences** | | |
|  | I enjoyed the prehab program. | “*I just kind of got a little enthusiastic after doing it for a few days and got a little keener*.” |
|  |  | “*In the beginning [the exercises were difficult], yes but not anymore, no. As you progress during the weeks and that, right now I feel great and I love doing them. And they get easier as you go along*.” |
|  |  | *“Nothing hindered me, I just kind of got a little enthusiastic after doing it for a few days and got a little keener.”* |
|  |  | "*Well just that I thought it was probably very positive to do and hopefully I see benefits down the road sometime*." |
|  |  | "*No, I don’t think so. I think if I had to do it again, I’d like what I’m doing right now*" |
|  |  | *“For the cardio, I have some nice walks that I quite look forward to doing.”* |
|  |  | "*Not too much. I liked the exercises and I did them three times a week except for a couple of exercises there that I didn’t get around to. But other than that, I enjoyed them*." |
|  |  | *“Actually, most days I felt good.”* |
|  |  | *“So yeah, I felt better after walking. I get very stiff if I’m sitting too long, so yeah, I felt better afterwards.”* |
|  |  | "*Well I think I felt good because I felt I was doing something to help myself through the surgery. I felt good about that*." |
|  |  | "*No, like I said, I feel great after. It’s a sense of accomplishment. So I’m one step closer to the operation and one step more prepared for the operation*." |
|  |  | "*Good. I enjoyed it. It didn’t bother me to be doing it at all and I knew there was a reason for it, to help my breathing and what muscles I have left. No. Just energetic and that’s about it. And I liked it*." |
|  |  | "*Yes. And on weekends we’d walk around in a mall or something, but I really liked going around the track at the indoor soccer field. You can’t use it on weekends because there are always other sports things going on there, so we just walk around in the mall or something because it’s been too cold to walk outside. But I miss it whenever we can’t. Yeah, I like it. I like walking*." |
|  |  | "*It was fun. It’s weird, but yeah, it was fun. I was able to do it like I said, on my own for the ones that I can do. And I guess overall you’re feeling better because you are doing some exercise rather than sitting and watching TV. From that aspect of it, it takes your mind off of why you’re doing them because like I said, the exercise program, it helps in that way that you’re not thinking about the surgery or anything else. You’re focusing more on the exercises and how they’re making you feel better, quitting smoking or whatever*." |
|  |  | "*I found I felt better after doing cardio, as opposed to when I didn’t. It is good to do, for sure. Yeah, no it all felt good to do, for sure*." |
|  | Having experience with exercise in past helped me complete the prehab program. | “*I think having a history of exercising previously would help a lot*” |
|  |  | "*Well, you have to have a little bit of strength because I noticed with my sister some of the exercises I had been doing, I could do 20, and she had to stop after five or something like that. So you do need some—*" |
|  |  | "*Well you have to learn how to do the exercises and so you kind of have to know what’s it’s supposed to stretch and it’s because the name of the exercises tell you what you’re stretching or trying to build up the strength in. I think there’s just some skill in knowing that okay, this is a bicep muscle. So if I do it like this, this is what I’m feeling. But it’s not a lot of skill, I don’t think*." |
|  |  | "*No, I don’t think so. I think that was pretty good. The chair squats and that were some of the things I did before. And the calf raises and stuff like that I’ve done before*." |
|  |  | "*But I did always like walking, but other exercise I never, ever did. Well I did at the breathing at the hospital here in Cornwall. I did exercise there and I really liked it*." |
